# Abnormal network flow detection based on application execution patterns from Web of Things (WoT) platforms

**DOI:** 10.1371/journal.pone.0191083

**Published:** 2018-01-19

**Authors:** Young Yoon, Hyunwoo Jung, Hana Lee

**Affiliations:** Department of Computer Engineering, Hongik University, Seoul, South Korea; Dalian University of Technology, CHINA

## Abstract

In this paper, we present a research work on a novel methodology of identifying abnormal behaviors at the underlying network monitor layer during runtime based on the execution patterns of Web of Things (WoT) applications. An execution pattern of a WoT application is a sequence of profiled time delays between the invocations of involved Web services, and it can be obtained from WoT platforms. We convert the execution pattern to a time sequence of network flows that are generated when the WoT applications are executed. We consider such time sequences as a whitelist. This whitelist reflects the valid application execution patterns. At the network monitor layer, our applied RETE algorithm examines whether any given runtime sequence of network flow instances does not conform to the whitelist. Through this approach, it is possible to interpret a sequence of network flows with regard to application logic. Given such contextual information, we believe that the administrators can detect and reason about any abnormal behaviors more effectively. Our empirical evaluation shows that our RETE-based algorithm outperforms the baseline algorithm in terms of memory usage.

## Introduction

In this paper, we aim to develop a novel technique for detecting abnormal situations proactively at the network monitor layer during runtime, based on the execution patterns of Web-based applications. However, gaining the awareness of the Web-based application behaviors at the network layer has been a non-trivial task. Asking every single independent server for their application execution patterns is not feasible.

Recently, new opportunities for gaining application awareness are arising, as Web of Things (WoT) platforms such as IFTTT [1] and Zapier [2] are emerging. These platforms came into service to support flexible composition of applications with various things connected to the Web. A user can easily select an application component from a pool of building blocks such as sensor information, actuation functions and data services to create and deploy personalized applications. A growing number of independent vendors are onboarding the WoT platforms in order to provide these building blocks [3]. We can reasonably expect more Web applications to be created through such WoT platforms because of the ease of development. We think inquiring WoT platforms for the application behaviors is a more feasible approach compared to the method of inquiring every individual Web server.

**Fig 1:**
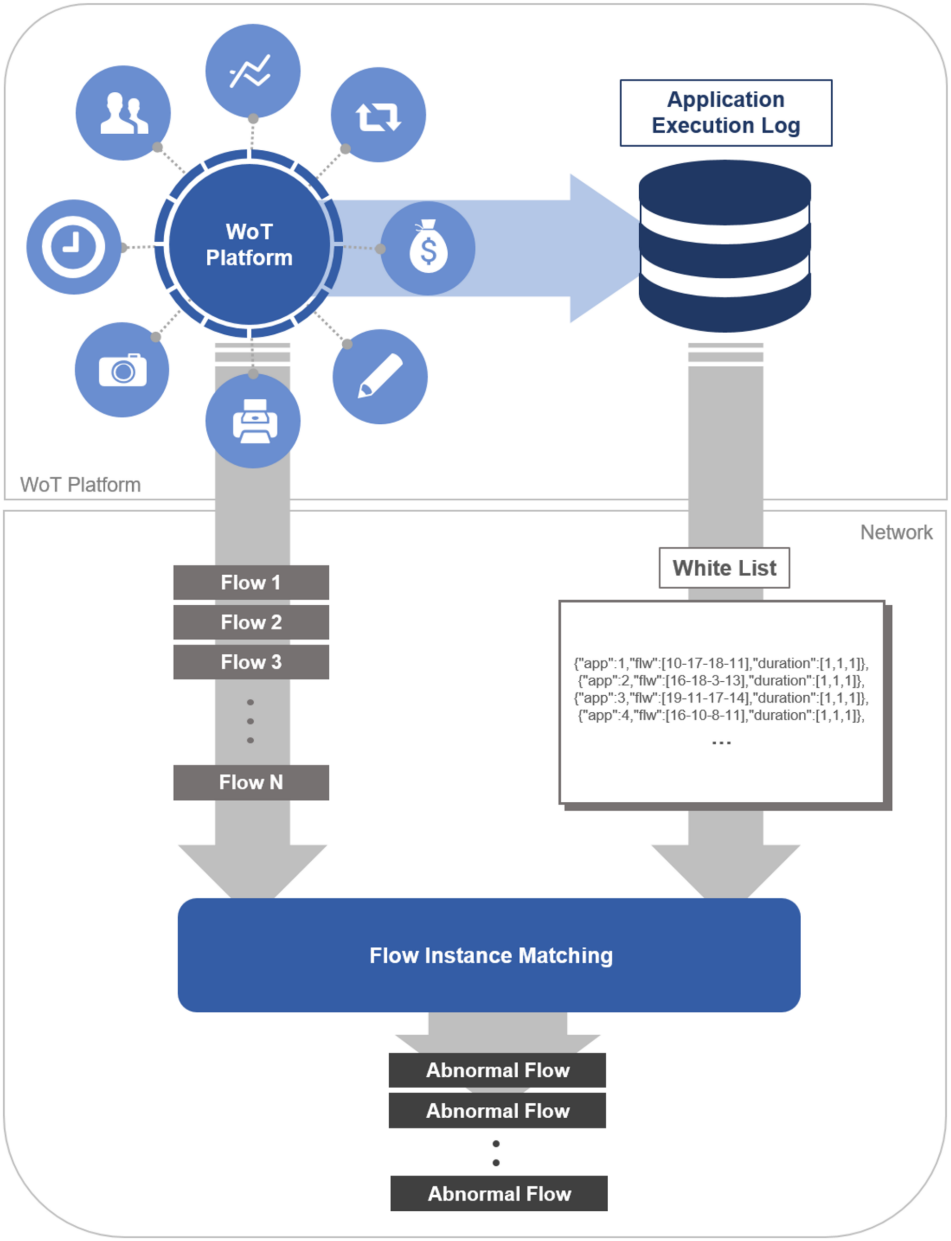
An overall framework for application-aware abnormal network flow detection.

Given the access to the application execution patterns on the WoT platforms and the underlying network systems where those WoT platforms run on, we aim to identify abnormal behaviors at the network monitor layer during runtime, as illustrated in Fig 1.

In this paper, we define an execution pattern of an application as a time sequence of Web service invocations. Web service is categorized into either a *trigger* or an *action* in WoT. A trigger is either a publication of some information or a signal that an action (actuation) took place. An action is a task to be executed whenever a trigger is fired. For instance, suppose a user wants to be notified when it rains. Using the composition tools of IFTTT or Zapier, the user, for example, can select a weather forecast service as a trigger and a push alarm service as an action. We assume that the WoT platforms log execution traces for every composed application and profile the average behavior into a time sequence.

Our system translates the time sequence of trigger and action executions to a time sequence of *network flows*. A *network flow* is a traffic information between two communicating endpoints [4]. Such information can be used for traffic engineering and security monitoring [5, 6]. Our system compiles a whitelist out of these time sequences of network flows. Our system collects the time sequences of flow instances (i.e., network flow events) and checks if any of these time sequences does not match a pattern in the whitelist. Flow instances that do not conform to the whitelist are regarded as an abnormal events, and they are placed in a watchlist for further review. The abnormal events may reflect performance disruptions at the WoT platform or a security breach.

**Fig 2:**
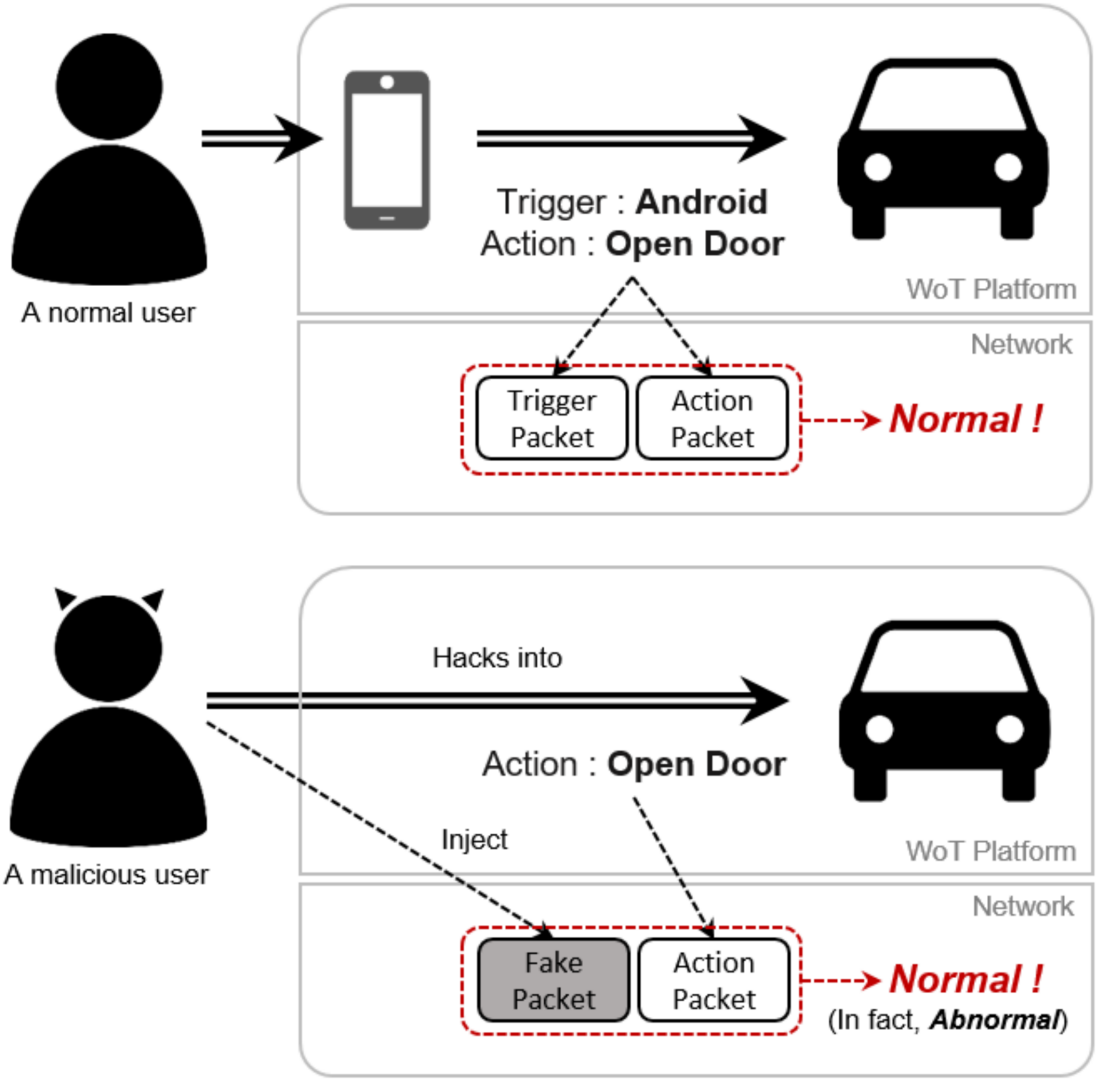
An example of security breach that occurs without being noticed by the security monitoring agents at the WoT platform and at the network layer.

We believe that this new method is a significant enhancement to the previous approaches. Abnormal changes can be detected through analysis of network packets [7]. However, these techniques can report many false alarms, especially when they are not aware of the application logic and behaviors. On the other hand, a stealth execution of a compromised application may go unnoticed by both the monitoring agents at the network layer and the platform unless they work in concert. For example, as shown in Fig 2, suppose a car driver composed and deployed an application that can automatically start engine when he/she is in close proximity of the car. A malicious user may compromise this application and start the engine even without being close to the car. This malicious user may inject a flow instance to the network layer and pretend that the engine start was a planned reaction to a valid trigger. With the whitelist of valid execution patterns expressed in network flows and the cooperation between the monitoring engines at both the network layer and the platform, the aforementioned problems can be resolved.

In this paper, we dive into the details of the implementation of this system. We focus more on the algorithms for matching real-time flow instances against the whitelist. The first algorithm we refer to as Whiplash is a base-line, brute-force algorithm that matches every populated partial time sequence against an entire whitelist. The second algorithm we refer to as TimedRETE matches a sequence of network flow instances against the whitelist stored in an applied RETE data structure [8].

Our key contribution can be summarized as follows. We present a novel research work that suggests to distinguish between normal and abnormal behaviors at the network layer based on a whitelist compiled out of the application execution patterns from WoT platforms. The detailed presentation of our contribution is structured as follows. First, we provide several definitions and assumptions necessary for expressing a whitelist. Second, given a whitelist, we present how it is leveraged by two algorithms. Third, we show the results from comparative experiments to reveal the pros and cons of our new algorithms. Fourth, we put our work in the context of various related works. Finally, we list possible future research directions and conclude.

## Definitions and assumptions

In this section, we design the overall system that processes real-time flow instances to determine whether they are abnormal according to the whitelist generated from the execution patterns available on WoT platforms.

A whitelist is a list of valid application execution patterns. Each entry in a whitelist is defined in terms of the network flows with the following pairs of information.

“Application ID”: Number,
“Network Flows”: [Numbers],
“Time delays”: [Numbers]

The attribute “Application ID” is an identifier of a WoT application. The attribute “Network Flows” is an ordered list of network flow identifiers. As mentioned earlier, a network flow is a network footprint that is generated when executing a WoT application. The flow instance contains information such as IP addresses and ports of the endpoints, the volume of the flow in terms of the number of packets, types of the application and the protocol used. The attribute “Time delays” is an ordered list of time delays between the occurrence of network flows listed under the “Network Flows” attribute. For instance, the following whitelist means that an application with an ID of 1 causes network flows 5, 7, 4 and 8 to occur in order, and the time delays between the occurrence of network flows will be commonly 1.

“Application ID”:1,
“Network Flows”:[5, 7, 4, 8],
“Time delays”:[1, 1, 1]

We refer to the occurrence of a specific network flow as a *flow instance*.

A WoT application is a combination of trigger and action services. A WoT platform maintains a REST endpoint that accepts a trigger from trigger services. The WoT platform invokes the REST endpoint of an action service that is planned to be executed upon receipt of a trigger event. Therefore, every application execution should generate a sequence of flow instances between the involved trigger/action services and the WoT platform.

These flow instances can be detected in real-time by tapping into the network with deep packet inspection (DPI) appliances, which can inspect up to 40 giga bits of packets and identify 40 million concurrent flows per second. [9, 10]. However, note that the packet inspection devices cannot identify the exact application workflow that caused a detected flow instance. At the network layer, multiple candidate applications match a detected flow instance, especially when flow instances are interleaved. Therefore, we require the WoT application to confirm which application corresponds to the detected flow instance, as it contains not only the complete information about the individual application logic and also the execution logs. Despite the complete application information available at the WoT platform, it is the flow instance monitoring agent at the network layer that first detects the signs of abnormal behavior. As introduced earlier, a user with malicious intent can inject fake flow instances to pretend that an action was executed as planned. Such covert activity cannot be detected solely at the WoT platform level.

However, deploying the monitoring appliances to the network on which a real WoT platform resides is not yet in the scope of this research work. Instead, we assume that a WoT platform is given and we devise a simulator that can synthesize various whitelists and generate simulated time sequences of flow instances.

Our system depends on the WoT platforms to profile the execution pattern of every application. We assume that an error bound for the duration between any two flow instances is given. The technique for profiling the performance of WoT applications precisely is an orthogonal issue. However, it is an interesting subject for future research. As another line of possible future work, we can account for the applications that implement more complicated conditional statements and loops, as seen typically in enterprise workflows. However, according to our investigation, major state-of-the-art WoT platforms such as IFTTT and Zapier just support applications to be composed with up to 2 services.

In the following section, we present the algorithms for detecting abnormal situations given a whitelist.

## Design of the flow instance matching algorithms

### The Whiplash algorithm

Whiplash is a simple algorithm that searches through an entire whitelist. Whenever a new network flow instance appears, Whiplash iterates through the whitelist to detect a normal sequence of flow instances.

Whiplash utilizes a PatternQueue which is a queue containing network flow instances. Whenever a flow instance is detected, Whiplash adds it to the end of the PatternQueue.

**Fig 3:**
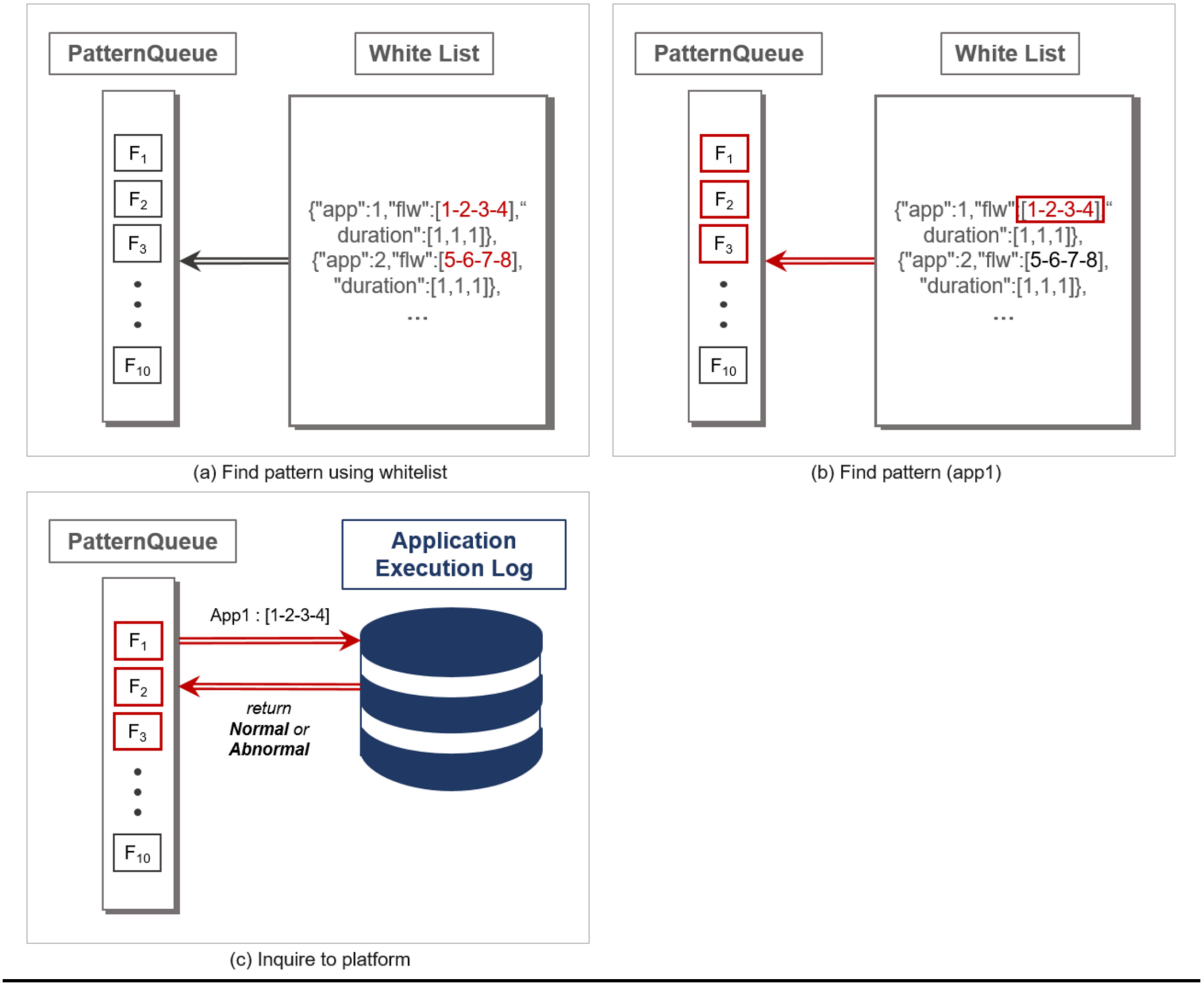
An example of sequentially updating PatternQueue with network flow instances being generated in real-time.

**Fig 4:**
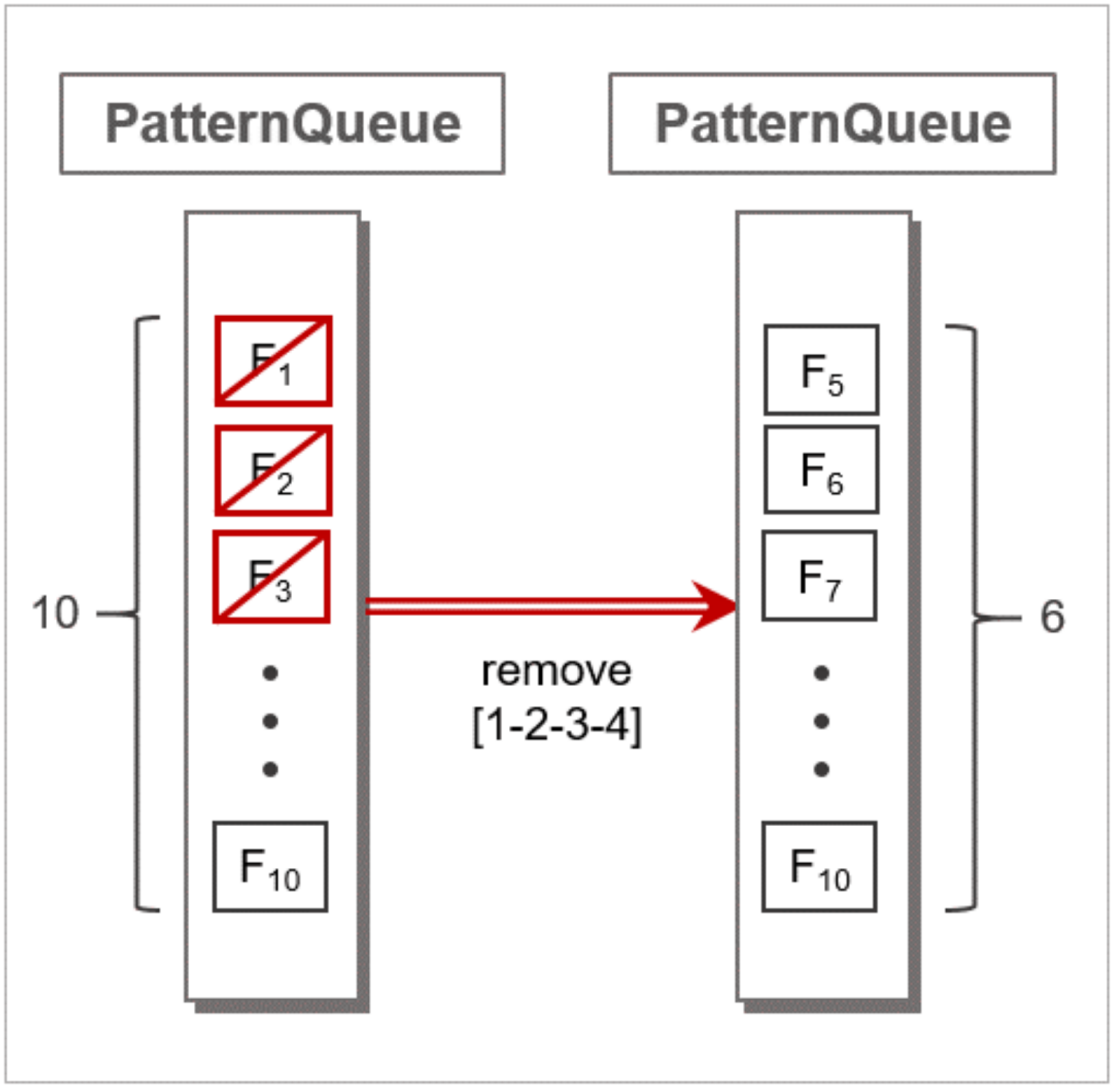
An example of matching time sequence pattern and clearing it from PatternQueue.

As soon as the flow instance gets added to the PatternQueue, matching the current flow instances against the entries in the whitelist takes place. For every entry of the whitelist, Whiplash searches for a matching sequence of flow instances in the PatternQueue, as shown in Fig 3(a) and 3(b). Note that Whiplash may return multiple candidates that match a whitelist entry. In such a case, Whiplash forwards the application ID of the matched whitelist entry and the actual time sequence of flow instances to the WoT platform. In return, the WoT platform confirms whether the services involved in the application were actually executed as specified in the time sequence, as shown in Fig 3(c). If a candidate match is confirmed, Whiplash moves on to the next whitelist entry. If the flow instances are confirmed to be valid footprints of an application, they are immediately removed from the PatternQueue. The normal time sequence of network flow instances found by the Pattern Search method is removed from the PatternQueue, as shown in Fig 4.

None of the candidate matches may be confirmed to be normal by the WoT platform. This does not necessarily mean that these candidate matches potentially reflect an abnormal situation. This is because, these candidate matches can be related to other whitelist entries. Here is how Whiplash collects potentially abnormal flow instances. For every network flow *F*, Whiplash first finds the maximum duration of a full time sequence that starts with *F*. Then Whiplash periodically sweeps through the PatternQueue to identify any flow instance that resided in the PatternQueue for more than maximum duration. These flow instances are removed from the PatternQueue and placed into the watchlist for further review, since we can suspect these to be abnormal.

Whiplash may easily suffer a premature eviction of perfectly normal flow instances, especially when the next PatternQueue sweeping cycle starts even before the entire whitelist is checked. We can let Whiplash wait until the entire whitelist entries are checked. However, this may overload PatternQueue. Apparently, we should employ a better approach to match time sequences against a whitelist. In the following section, we present the RETE-based algorithm.

### The TimedRETE algorithm

In this section, we design TimedRETE algorithm. This algorithm addresses the issue of Whiplash checking the entire whitelist for every possible time sequence in the PatternQueue. We extend RETE [8], a graph-based algorithm for matching real-time events against specific patterns. A number of modern complex event processing (CEP) systems are based on the RETE algorithm [11–13]. However, these CEP systems come short in providing the means to express the interest in detecting all patterns that are different from a set of normal patterns. Moreover, storing whitelist of application execution patterns in a RETE network has not been studied in depth. This prompts us to design a new RETE-based algorithm.

In the following, we present TimedRETE. We explain how it stores a whitelist of network flow execution patterns into a RETE network. We show how TimedRETE traverses through the RETE network to identify normal and abnormal patterns.

#### Construction of a RETE network

**Fig 5:**
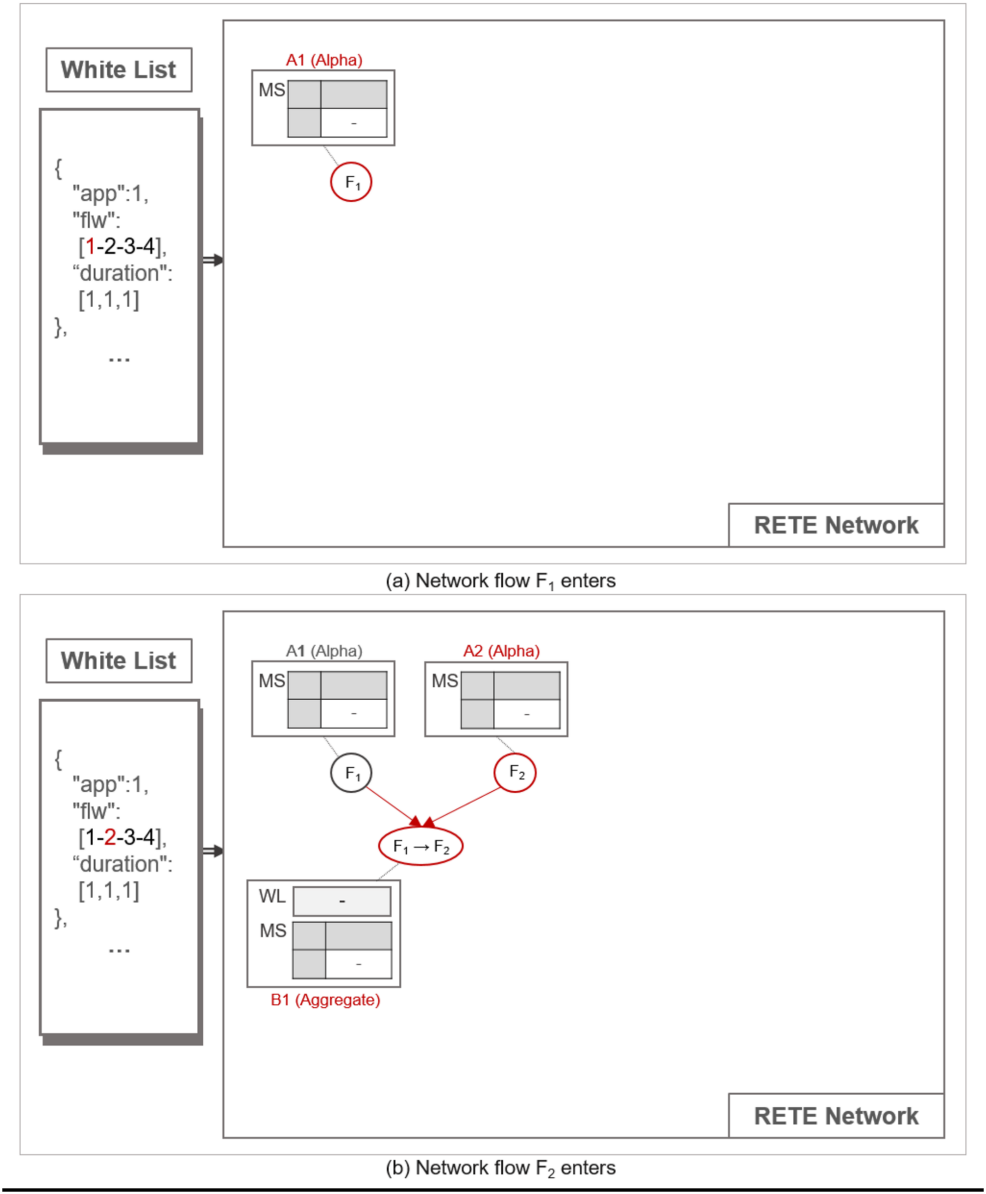
The process of RETE network construction.

**Fig 6:**
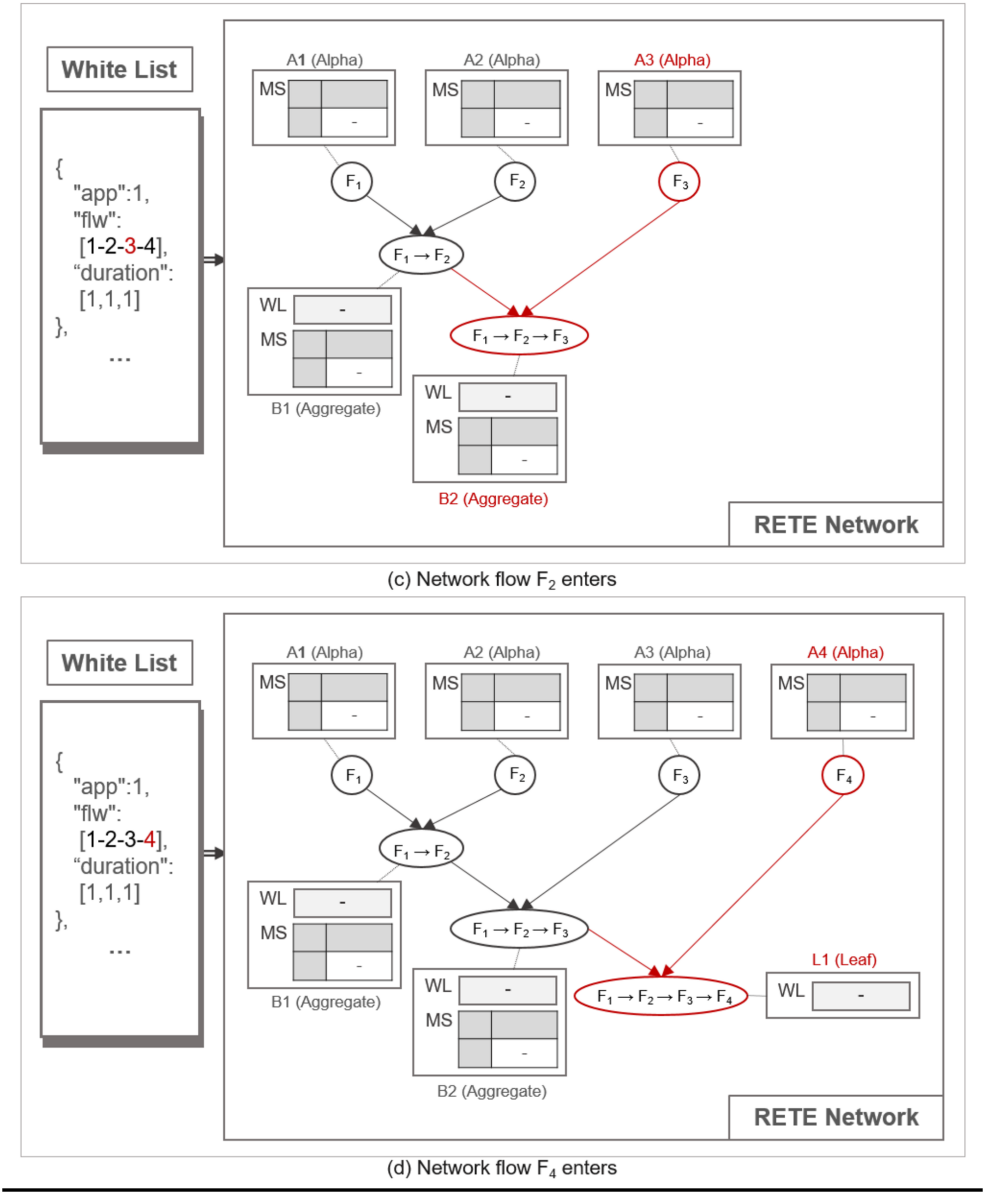
The process of RETE network construction.

TimedRETE stores a whitelist obtained from a WoT platform into a network of alpha, aggregate and leaf nodes. Alpha node stores a single network flow and matches incoming flow instance. Aggregate node correlates flow instances from alpha nodes. Leaf node stores the last network flow in the whitelist entry. We denote the alpha, the aggregate and the leaf node as *A*, *B* and *L*, respectively. Given a whitelist entry, TimedRETE iterates through the sequence of network flows and associates them with alpha, aggregate and/or leaf nodes. If an alpha node does not exist for a given network flow, TimedRETE creates a new one (*A*_1_). For instance, as shown in Fig 5(a), an alpha node for the network flow with ID of 1 is newly created (*F*_1_), which is added to the root of the TimedRETE network. TimedRETE allocates an aggregate node for a subsequent network flow in the sequence and then correlates it with the previous network flow. For example, as shown in Fig 5(b), TimedRETE adds a new alpha node (*A*_2_) for the network flow with ID of 2 in the sequence (*F*_2_). Then TimedRETE creates the aggregate node (*B*_1_) that is connected to alpha nodes *A*_1_ and *A*_2_. This aggregate node stores the information about the time delay between *F*_1_ and *F*_2_. In this example, TimedRETE continues to create the alpha node (*A*_3_) and an aggregate node (*B*_2_) for the subsequent network flow (*F*_3_), as shown in Fig 6(c). In this case, the new aggregate node is connected to *A*_3_ and *B*_1_. *B*_2_ stores the information about the time delay between *F*_2_ and *F*_3_. TimedRETE repeats this process until it encounters the last element in the sequence of network flows. TimedRETE creates a leaf node for the last network flow. For example, as shown in Fig 6(d), the alpha node (*A*_4_) for the network flow (*F*_4_) is created. This alpha node is followed by the leaf node (*L*_1_), which is connected to the previously created aggregate node *B*_2_ and *A*_4_. Finally, *L*_1_ keeps the information about the time delay between *F*_3_ and *F*_4_.

An aggregate node has two parents: *a trigger parent* and *an action parent*. A parent node is a trigger parent, if it is to forward an instance of a flow that precedes the flow of the action parent. For instance, as shown in Fig 5(c), *A*_1_ and *A*_2_ are a trigger parent and an action parent of *B*_1_, respectively. We call flow instances forwarded by a trigger parent and an action parent as *a trigger instance* and *an action instance*, respectively.

Nodes other than the alpha nodes keep a list of trigger instances waiting to be matched with a subsequent action instance within the duration bounds as specified in the whitelist. We refer to this list as WaitList (WL). Note that the WaitList for the action instance is maintained only in the aggregate and the leaf nodes. Every node except the leaf nodes keeps the match states of every flow instance it sent to the immediate child node. This match state is put in a table we refer to as MatchStates (MS). Each MatchStates entry is a 4-tuple, ($f_{x}^{i}$, *C*, *State*, *count*). $f_{x}^{i}$ denotes a flow instance *i* of the network flow *f*_*x*_, *C* denotes an immediate child node. *State* is one of following four match states of $f_{x}^{i}$ at *C*, INIT, PARTIAL_MATCH (P_M), FULL_MATCH (F_M) and NO_MATCH (N_M). *count* is the total number of responses from the child nodes about the match state of $f_{x}^{i}$.

Given the aforementioned notions, we explain how incoming flow instances are matched against the constructed RETE network containing the whitelist information, in the following section.

#### Pattern matching procedure

A flow instance traverses the RETE network as specified in Algorithm 1. TimedRETE computes the match states of the flow instance during the traversal. This algorithm can be explained best with a series of detailed examples illustrated in Figs 7, 8, 9, 10, 11, 12, 13 and 14.

**Algorithm 1:** RETE Network Traversal

**Input:** Flow instance *f*

1 **if** *Alpha node A receives f* **then**

2  Relay *f* to child nodes *C*;

3  **for** *child node c* ∈ *C* **do**

4   Add (*f*, *c*, INIT, *count* = |*C*|) to MatchState MS;

5 **if** *Aggregate node B receives f* **then**

6  **if** *f is trigger instance from parent P_t_* **then**

7   Add *f* to WaitList WL;

8   Relay *f* to child nodes *C*;

9  **if** *f is action instance from parent P_a_* **then**

10   **for** *trigger instance f′ ∈ WL* **do**

11    **if** *f′ matches f within duration bound* **then**

12     Relay instance *f*′ → *f* to child nodes *CS*;

13     **for** *child node c ∈ C* **do**

14      Add (*f*′ → *f*, *c*, INIT, *count* = |*C*|) to MatchState MS;

15     Change the state of *f* at *B* to *PARTIAL*_*MATCH* in MS of *P*_*t*_ and *P*_*a*_;

16     **if** *not the first match* **then**

17      Increment the count of *f* by 1 in MS of *P*_*t*_ and *P*_*a*_;

18     Break out of the loop;

19   **if** *no matching trigger instance found* **then**

20    Change the state of *f* at *B* to *NO*_*MATCH* in MS of *P*_*t*_ and *P*_*a*_;

21    Decrement the count of *f* by 1 in MS of *P*_*t*_ and *P*_*a*_;

22   **for** *every flow instance f_ ∈ MS whose count is 0* **do**

23    Change the state of *f*_ at *B* to *NO*_*MATCH* in MS of *P*_*t*_ and *P*_*a*_;

24 **if** *Leaf node L receives f* **then**

25  **if** *f is trigger instance from parent P_t_* **then**

26   Add *f* to WaitList WL;

27  **if** *f is action instance from parent P_a_* **then**

28   **for** *trigger instance f′ ∈ WL* **do**

29    **if** *f′ matches f within duration bound* **then**

30     Change the state of *f* at *B* to *FULL*_*MATCH* in MS of *P*_*t*_ and *P*_*a*_;

31     Decrement the count of *f* by 1 in MS of *P*_*t*_ and *P*_*a*_;

32     Break out of the loop;

33   **if** *no matching trigger instance found* **then**

34    Change the state of *f* at *B* to *NO*_*MATCH* in MS of *P*_*t*_ and *P*_*a*_;

35    Decrement the count of *f* by 1 in MS of *P*_*t*_ and *P*_*a*_;

**Fig 7:**
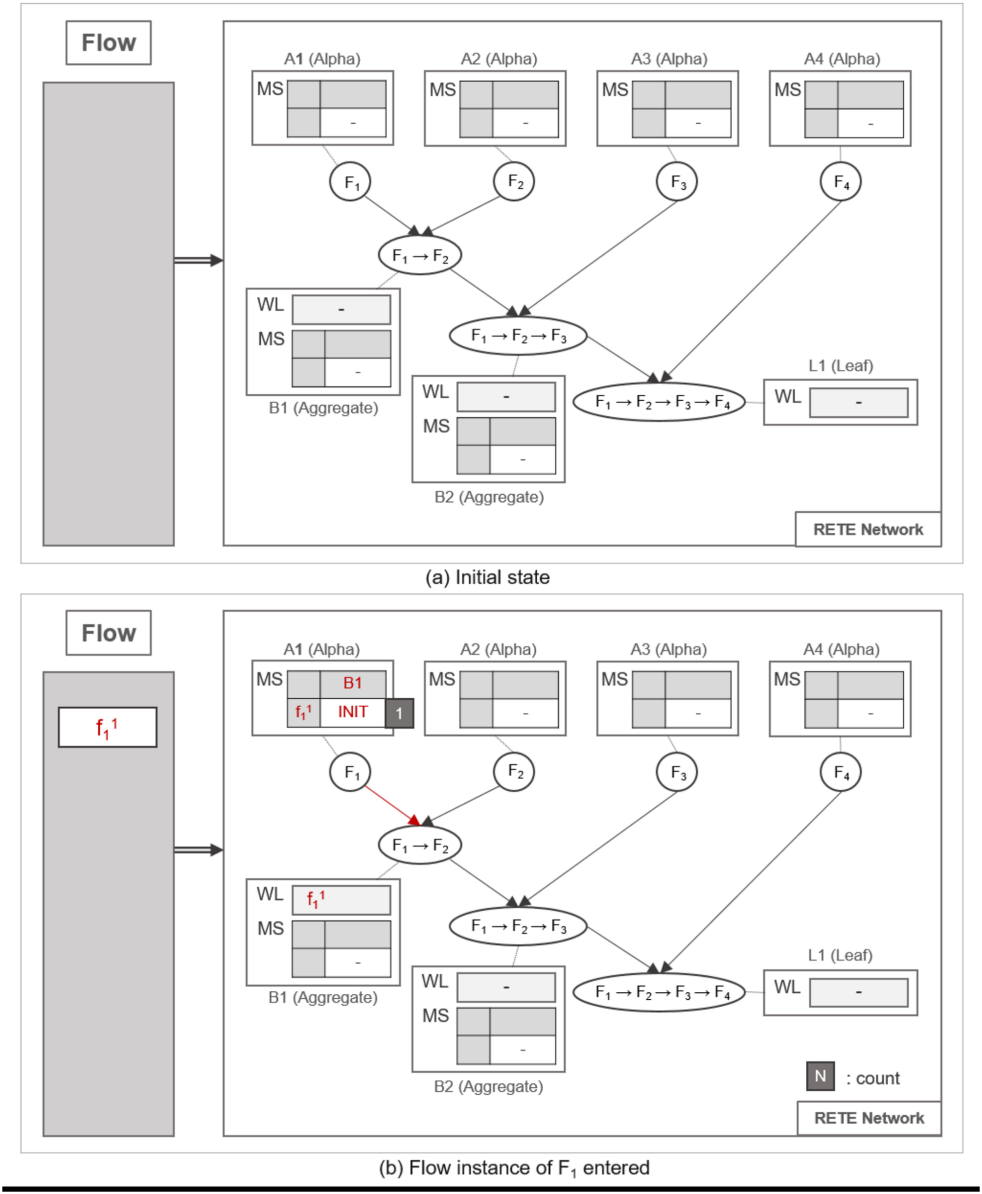
An example of locating alpha node and initializing its MatchStates upon receipt of a flow instance.

**Fig 8:**
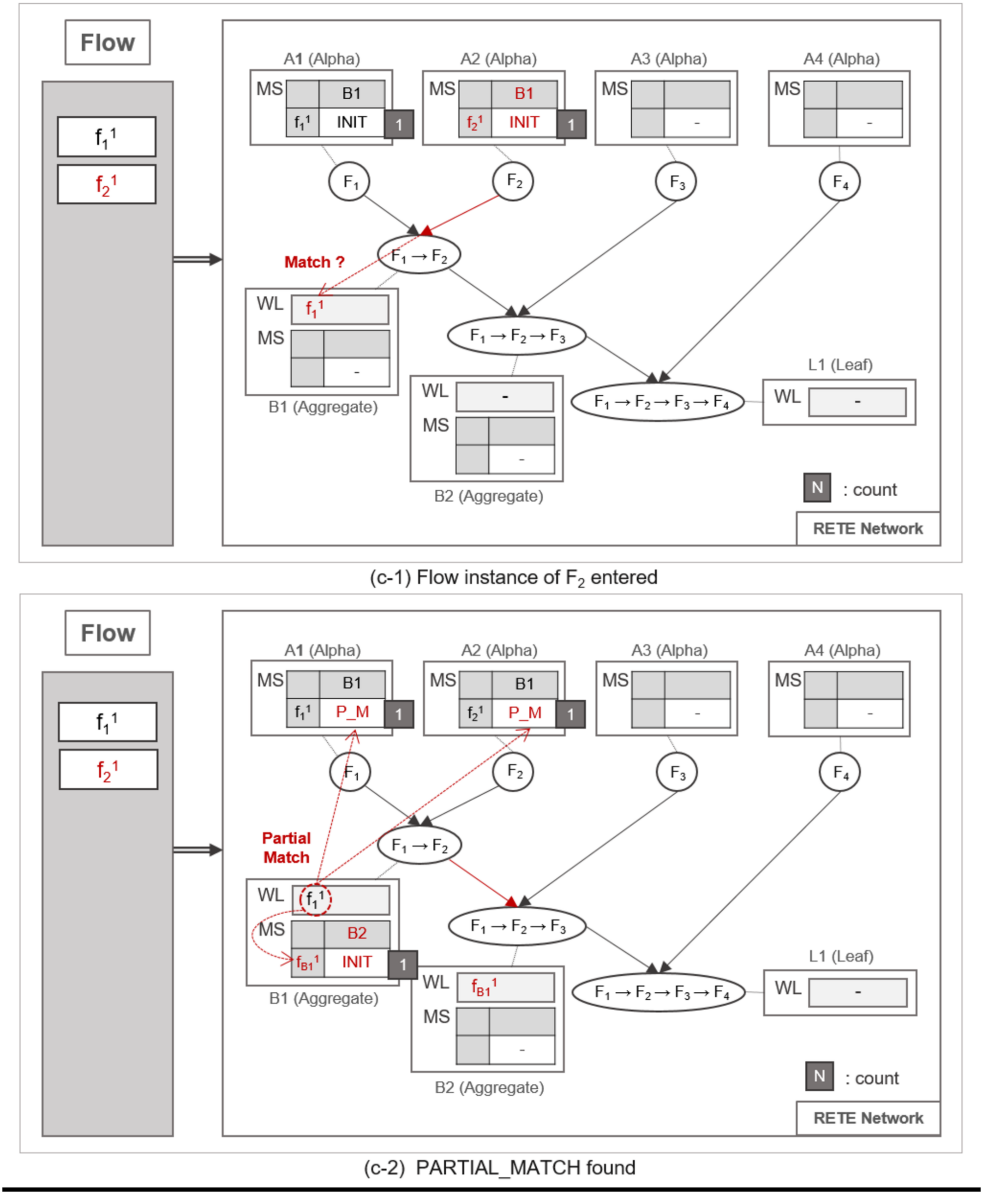
An example of detecting a partially-matched time sequence of flow instances.

**Fig 9:**
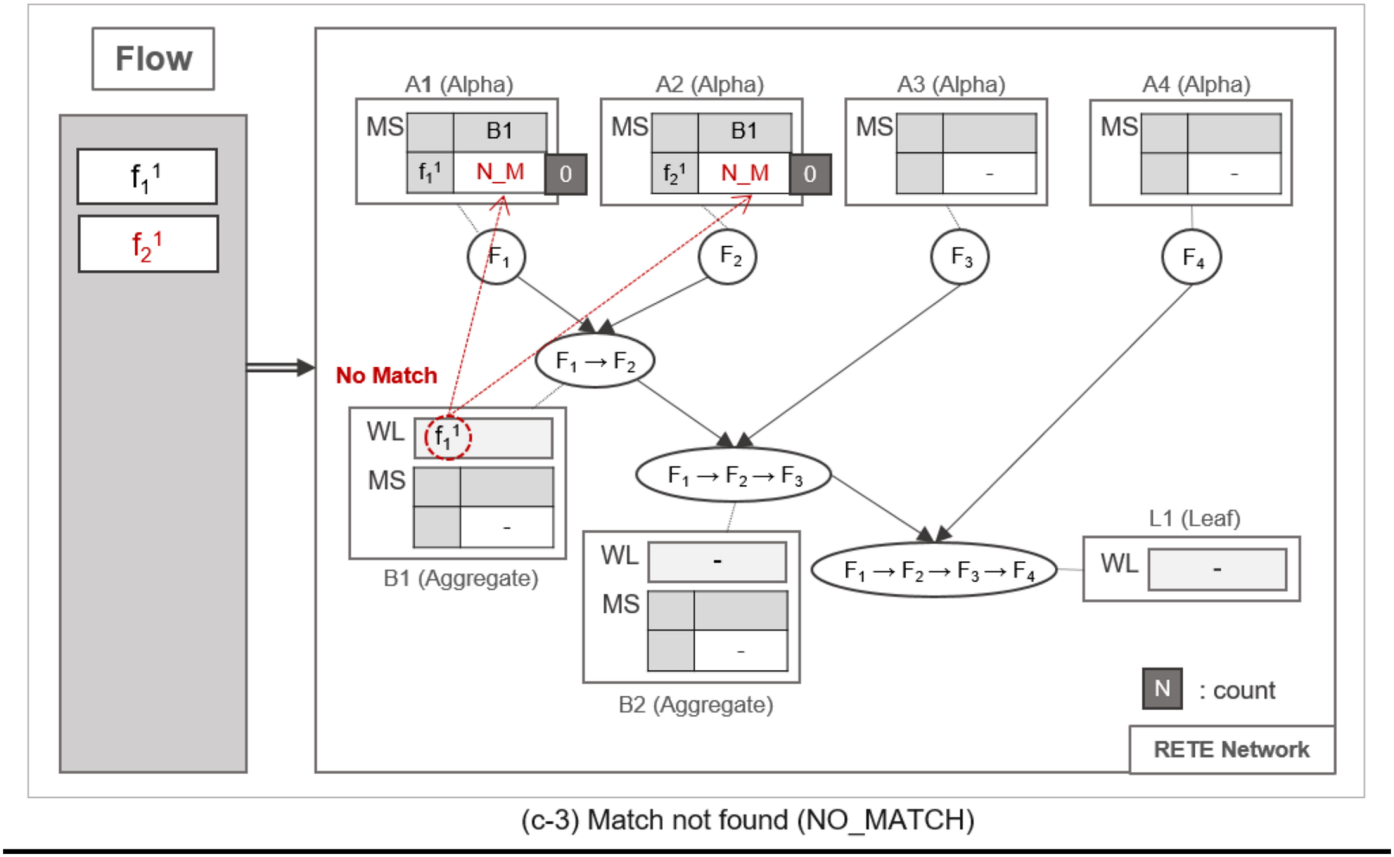
An example of finding no match when action instance enters an aggregate node.

**Fig 10:**
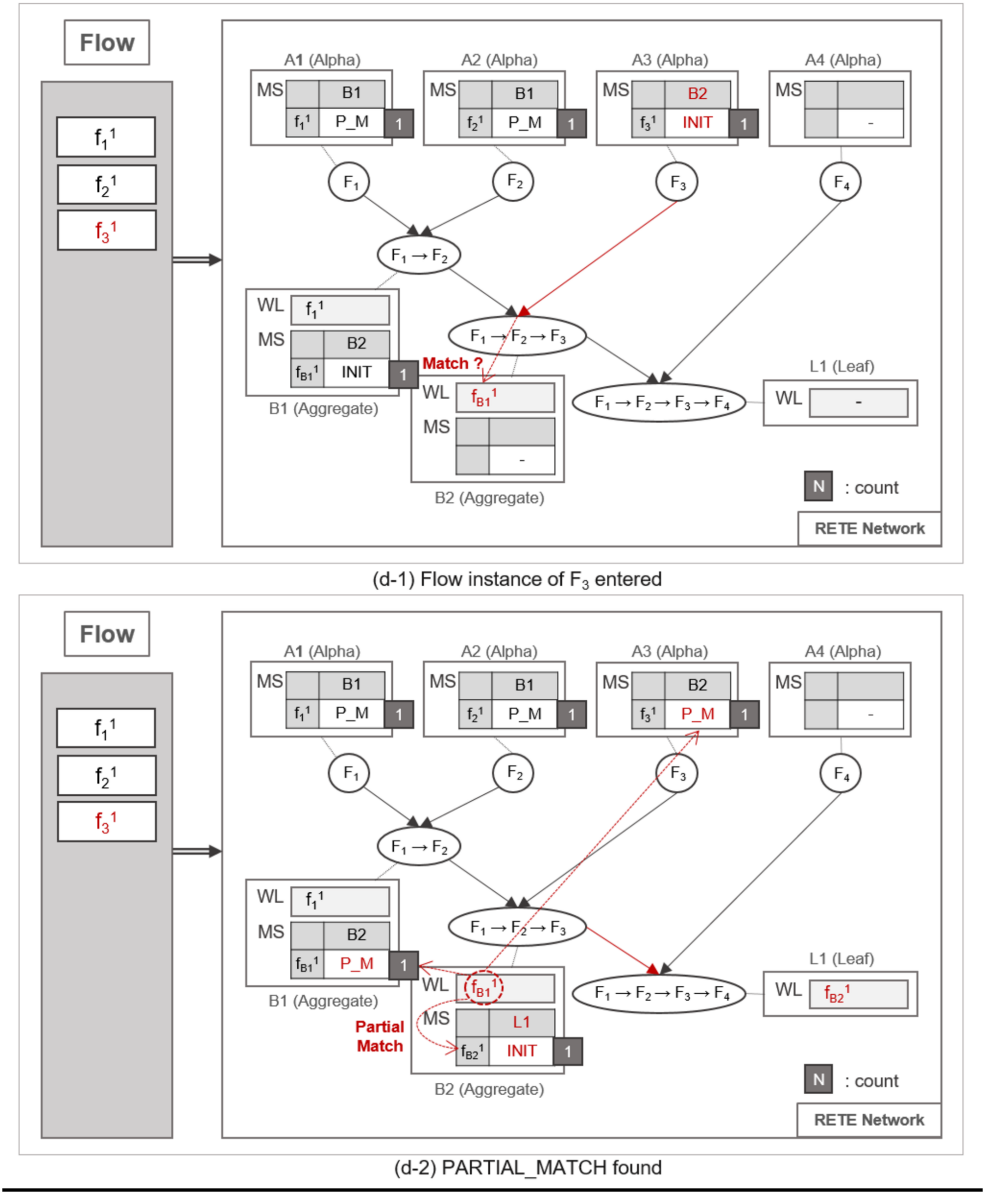
An example of repeated traversing until a leaf node is reached.

**Fig 11:**
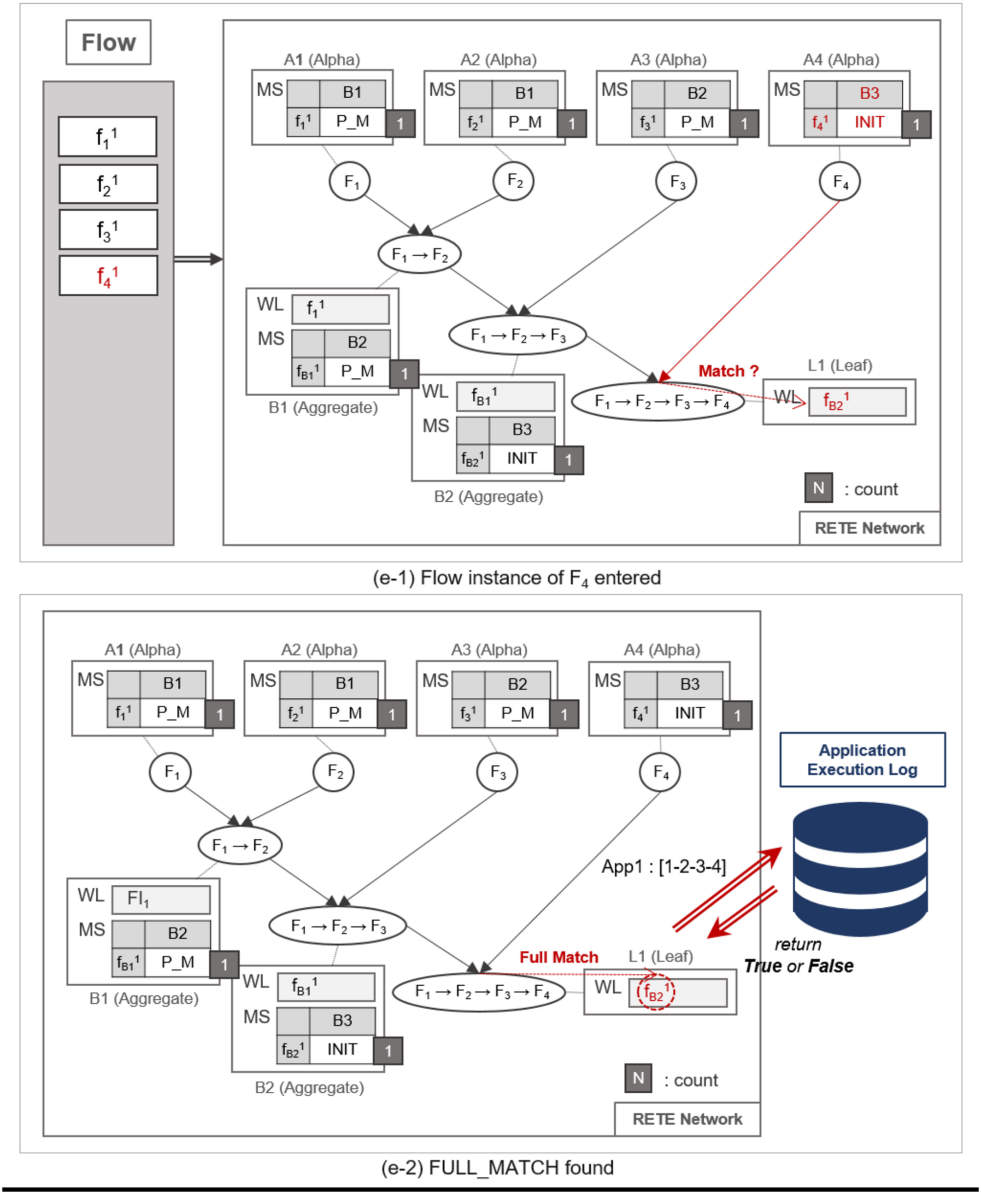
An example of a full match at the leaf node.

**Fig 12:**
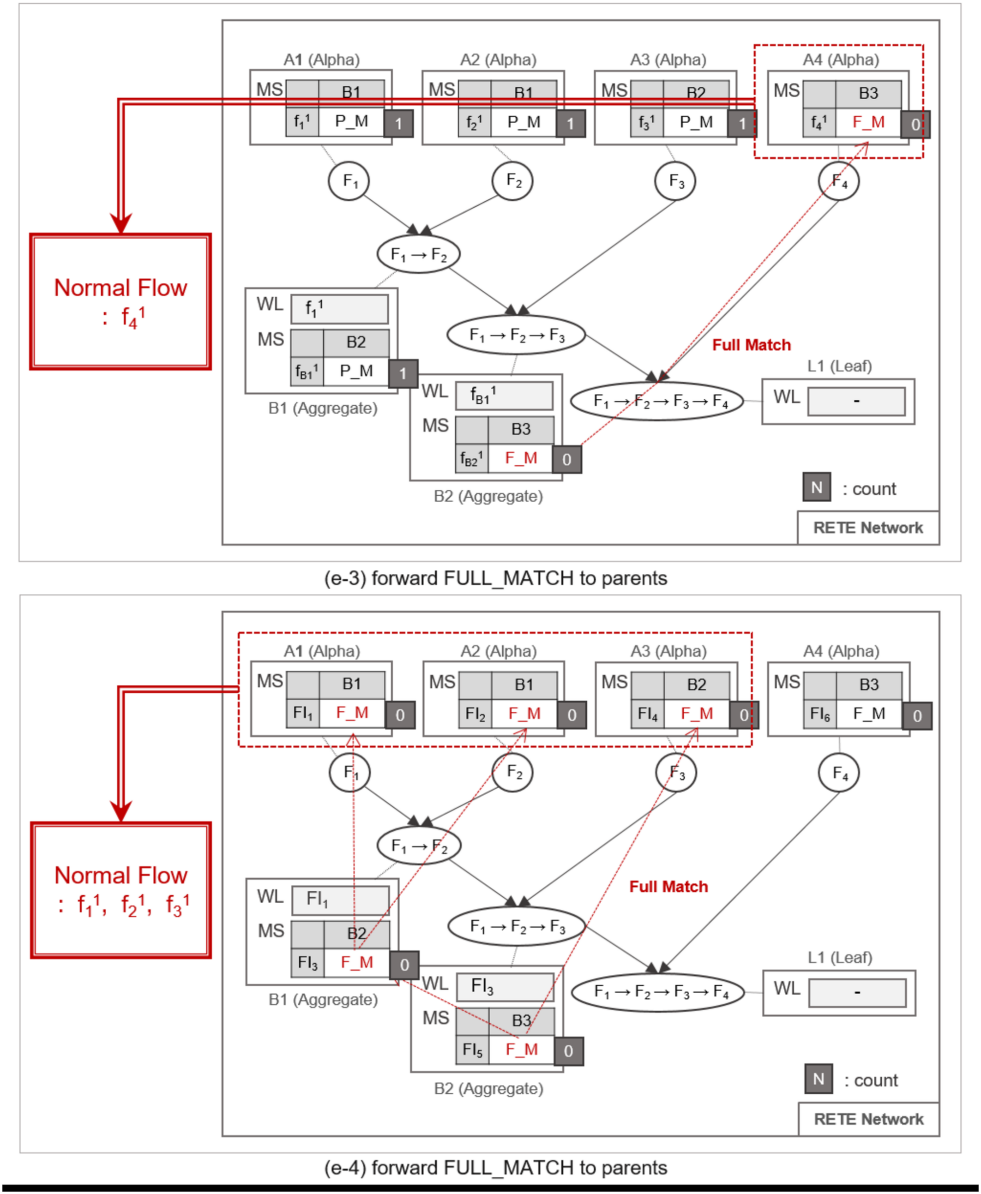
An example of identifying normal flow instances.

**Fig 13:**
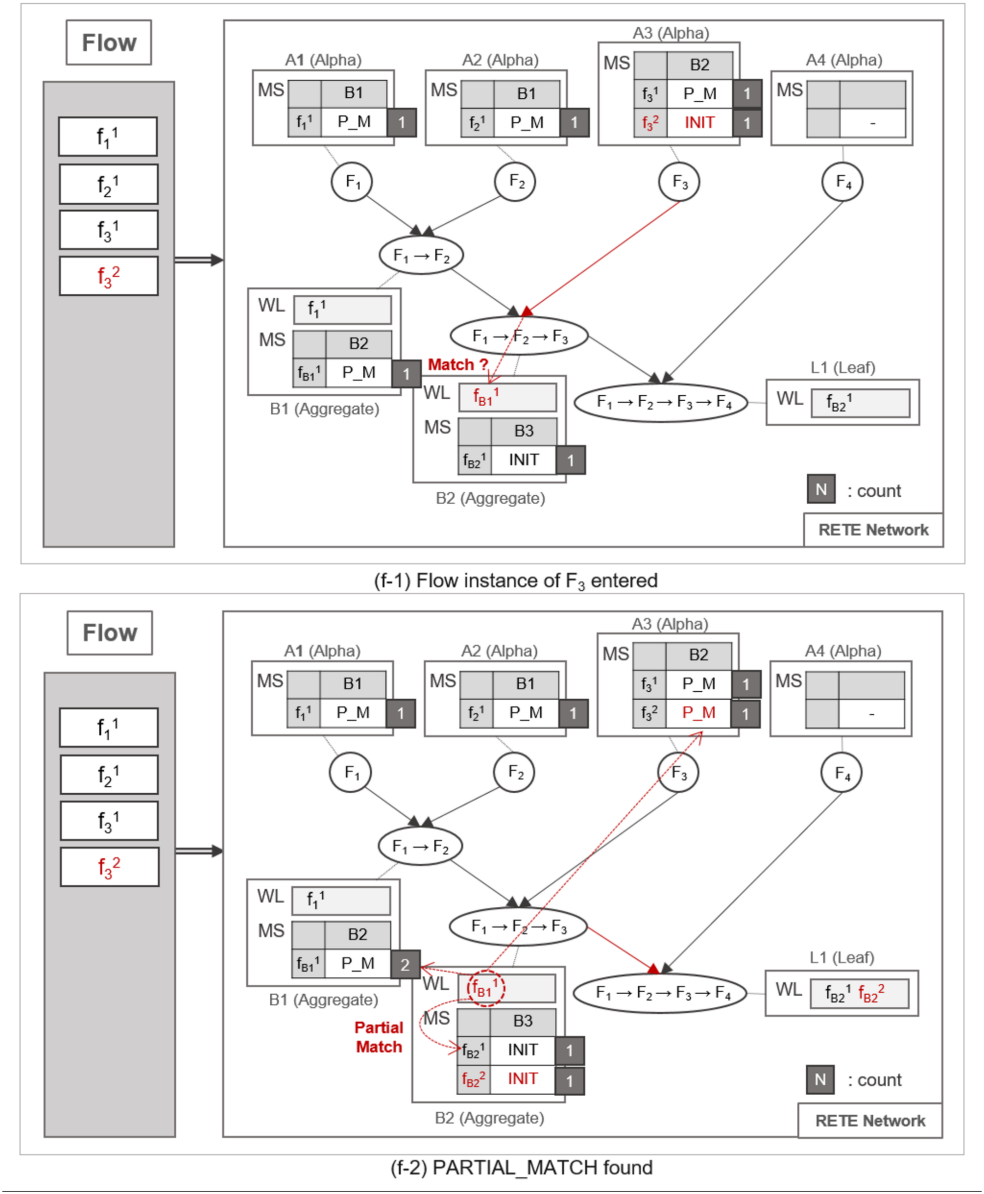
An example of utilizing count information for the retrieval of normal and abnormal flow instances.

**Fig 14:**
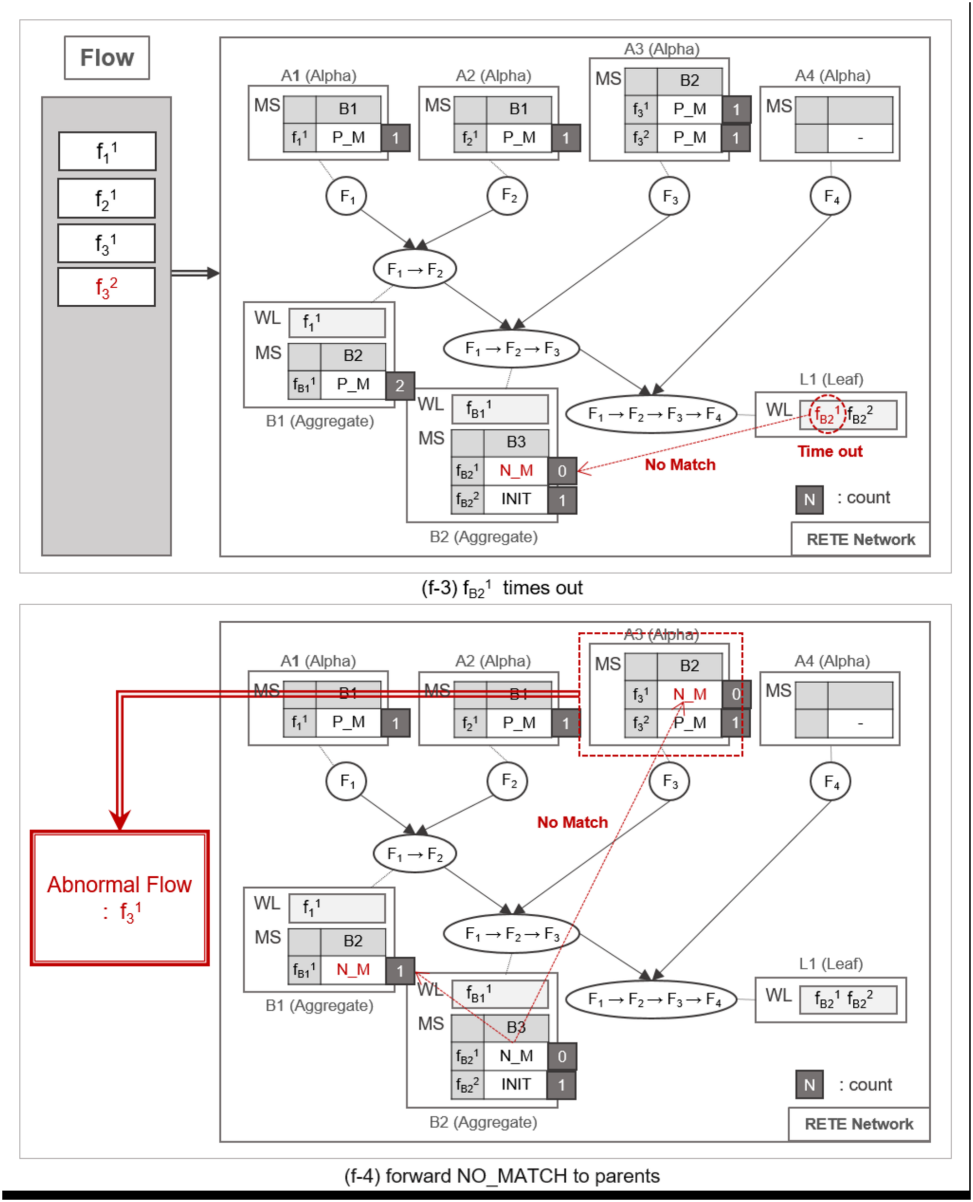
An example of utilizing count information for the retrieval of normal and abnormal flow instances.

Whenever a flow instance enters the RETE network for the first time, TimedRETE locates a corresponding alpha node. The located alpha node sets the match states of this flow instance at the child nodes to INIT. This alpha node adds the match state information to the MatchStates along with the count of the flow instance that is initialized to |*C*| which is the number of child nodes immediately succeeding *A*_1_. Suppose that the initial state of RETE Network is as shown in Fig 7(a). When a flow instance, $f_{1}^{1}$ enters this network, TimedRETE finds the alpha node *A*_1_ and sets the state of $f_{1}^{1}$ at the immediate child node *B*_1_ to INIT. Count of $f_{1}^{1}$ is initialized to 1 as there is only one immediate child node for *A*_1_. The state value of INIT indicates that the flow instance newly entered the system, and the process of the waiting for the matching subsequent instances has started. This match state information and the initial count value is added to MatchStates (MS) of *A*_1_. Subsequently, *A*_1_ forwards the $f_{1}^{1}$ to its immediate child node. If the child node receives the flow instance as a trigger instance, it adds the instance to the WaitList (WL) and waits for an action instance to be forwarded by the action parent, as shown in Fig 7(b).

When another flow instance $f_{2}^{1}$ enters the RETE network, TimedRETE finds alpha node *A*_2_ and repeats the MatchStates update procedure and forwards $f_{2}^{1}$ to its immediate aggregate node *B*_1_. *B*_1_ takes $f_{2}^{1}$ as an action instance as it was sent by *A*_2_, the action parent. Upon receipt of $f_{2}^{1}$, *B*_1_ iterates through the WaitList to see if there is any previous trigger instance that occurred prior to *F*_2_ within the duration bounds, as shown in Fig 8(c-1). If the trigger instances meeting the duration requirement are found, then *B*_1_ reports to its parent nodes that the match state for these trigger instances and the action instance should be PARTIAL_MATCH, as shown in Fig 8(c-2). Note that the count of $f_{2}^{1}$ is not updated if the PARTIAL_MATCH is the very first match found for $f_{2}^{1}$. If no trigger action satisfies the duration bounds, then we can regard the action instance to be unrelated with the trigger instances. The node reports to the trigger and action parent that the state of the action instance has to be NO_MATCH. This indicates that there is no relationship between the trigger instances and the action instance, as shown in Fig 9(c-3). Subsequently the *count* of $f_{2}^{1}$ is decremented by 1. The node that found a PARTIAL_MATCH state must forward the partially-matching time sequence to its immediate child node as a subsequent flow instance. For example, $f_{B1}^{1}$ is forwarded to *B*_2_, as shown in Fig 8(c-2).

Such matching and relay operation is repeated until a flow instance reaches a leaf node, as shown in Fig 10. When the flow instance $f_{3}^{1}$ enters the network as shown in Fig 10(d-1), MatchState information is initialized to INIT at *A*_3_. If a PARTIAL_MATCH is found at *B*_2_, the partially-matched time sequence is forwarded to the leaf node *L*_1_ as a trigger instance $f_{B2}^{1}$, as shown in Fig 10(d-2). $f_{B2}^{1}$ is placed in the WaitList of *L*_1_ waiting to be fully matched with the last flow instance, as shown in Fig 11(e-1). For example, if $f_{4}^{1}$ enters the network and traverses up to *L*_1_ it fully matches the partial time sequence $f_{1}^{1}$ → $f_{2}^{1}$ → $f_{3}^{1}$. Note that we cannot prematurely judge that the fully-matched time sequence to be normal, because the flow instances could have been invoked as a part of other applications. Therefore, along with the application ID, TimedRETE transfers the fully-matched time sequence to the WoT platform in order to get a final confirmation that the time sequence actually occurred according to application execution log as shown in Fig 11(e-2). If the time sequence is confirmed to be normal, *L*_1_ removes the flow instances from its WaitList and sends the state information FULL_MATCH to the parents, as shown in Fig 12(e-3). When the parent nodes receive either FULL_MATCH or NO_MATCH of a flow instance, *f*, these nodes decrement the *count* of *f* by 1. Immediately after the *count* value becomes zero, the node relays the state information to its parents along with the final match state information, as shown in Fig 12(e-4).

In the following section, we show how TimedRETE sweeps through the set of alpha nodes to retrieve normal and abnormal time sequence of flow instances.

#### Identifying normal and abnormal flow instances

The periodic process of identifying normal and abnormal flow instances is specified in Algorithm 2.

**Algorithm 2:** Retrieval of normal and abnormal flow instances

**Input:** A set of alpha nodes Δ

1 **for** *every t time* **do**

2  **for** *A* ∈ Δ **do**

3   **foreach** *flow instance f ∈ MatchStates of A* **do**

4    **if** *f*.*count* = = 0 **then**

5     **if** *there is a FULL_MATCH for f* **then**

6      *f* is a normal flow instance;

7     **else**

8      *f* is an abnormal flow instance;

9      Move *f* to the watchlist;

Algorithm 2 simply states that if the *count* value of a flow instance in the MatchStates of an alpha node is zero, and FULL_MATCH state is detected, then the flow instance is regarded to be a normal one. If there is no FULL_MATCH state while the *count* is zero, then the flow instance is considered to be an abnormal one, as shown in the example illustrated in Fig 14(f-4). Note that this checking procedure is based on the following theorem.

**Theorem 1**: If the *count* value is zero for a flow instance *f* in the MatchStates of an alpha node, than the possible state value of *f* has to be either FULL_MATCH or NO_MATCH.

Proof: We prove Theorem 1 by contradiction. Suppose that there can be a state value of INIT for *f* in the MatchStates of an alpha node while the *count* value is zero. *count* cannot be zero in this case as *count* is initialized to the number of immediate child nodes. Whenever PARTIAL_MATCH is found at the succeeding child nodes, *count* of *f* should be incremented. Therefore, the initial *count* of *f* can never become zero. Also, suppose there can be a state value of PARTIAL_MATCH for *f* in the MatchStates of an alpha node while the *count* value is zero. *count* of *f* should be incremented whenever PARTIAL_MATCH is found at the succeeding child nodes. Therefore, *count* cannot become zero unless all child nodes decrement *count* to zero by reporting either FULL_MATCH or NO_MATCH ■

Additionally, there should be only one FULL_MATCH for a flow instance across all alpha nodes, because of the initial assumption that a flow instance must be part of just one application execution. We guarantee this by getting the confirmation from the WoT platform base on its application execution log.

The *count* value of a flow instance plays a critical role of preventing TimedRETE from making a haste judgment on a flow instance. Suppose multiple trigger and action instances (e.g., $f_{B1}^{1}$, $f_{3}^{1}$ and $f_{3}^{2}$) arrive at the aggregate node *B*_2_, as shown in Fig 13(f-1) and 13(f-2). If $f_{3}^{1}$ does not match $f_{B1}^{1}$ within the duration bound, then *B*_2_ reports to *B*_1_ that no match is found for $f_{B1}^{1}$, as shown in Fig 14(f-3) and 14(f-4). Suppose, we change Algorithm 1 to relay the NO_MATCH state of $f_{B1}^{1}$ to the alpha node *A*_1_ and *A*_2_, without checking the *count* value. Then, at the next polling cycle to retrieve normal and abnormal time sequences according to Algorithm 2, some flow instances that turned out to be normal may be falsely identified as abnormal instances.

**Fig 15:**
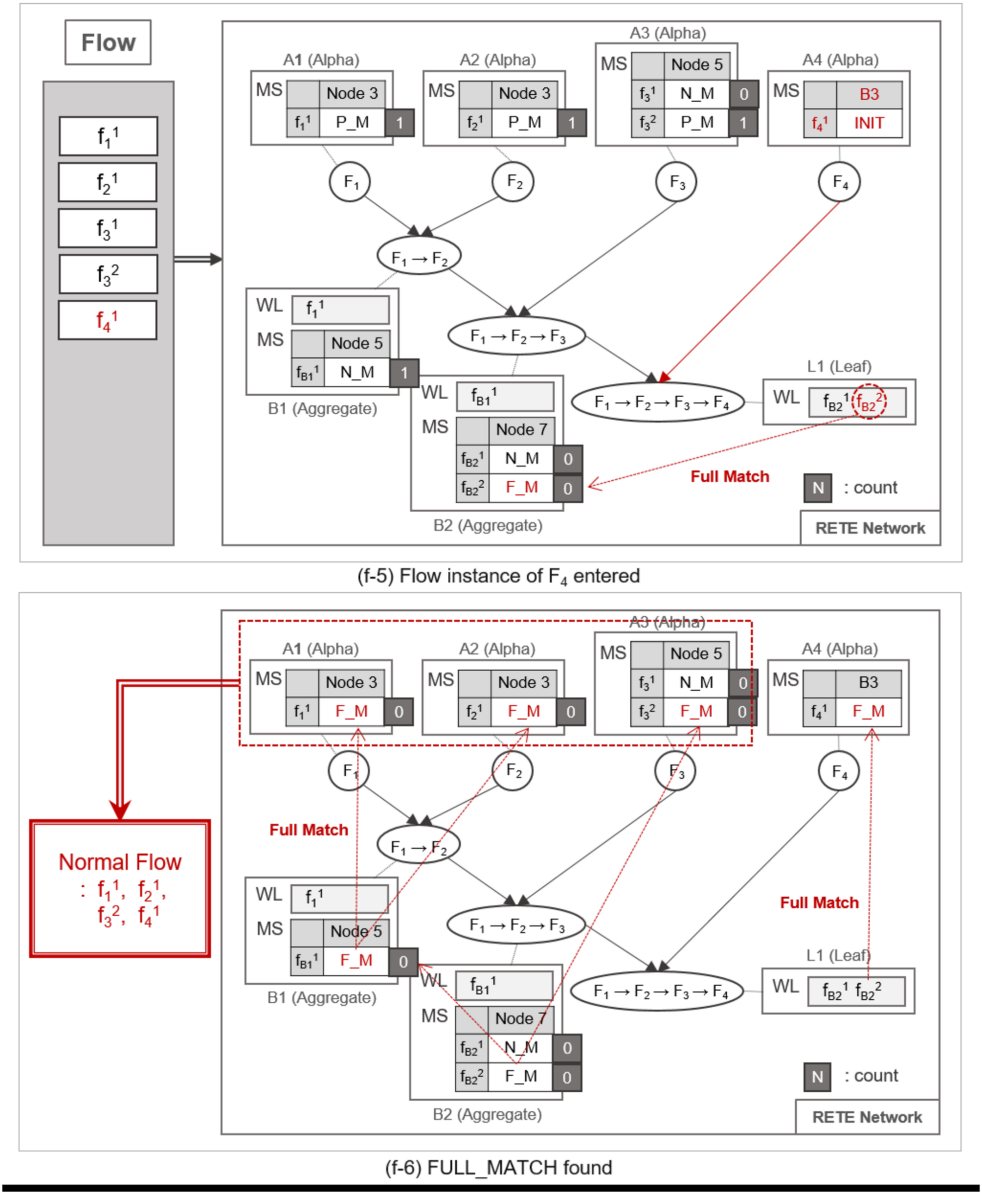
An example of utilizing count information for the retrieval of normal and abnormal flow instances.

As shown in Fig 15(f-5) and 15(f-6), the flow instances $f_{1}^{1}$ and $f_{2}^{1}$ turns out to match $f_{3}^{2}$ and $f_{4}^{1}$. Therefore, *count* can be seen as the number of pending PARTIAL_MATCH and FULL_MATCH to check for a flow instance.

#### Discussion

In this section, we mainly discuss the qualitative assessment of the performance of the two algorithms we presented.

Firstly, in case of Whiplash, it takes at most ${}_{f}P_{w}=\frac{w!}{(w-f)!f!}$ operations to find partial matches, where *f* is the number of flow instances currently in PatternQueue and *w* is the number of entries in the whitelist. The worst case is when there are only abnormal time instances. However, if WoT applications behave correctly, then the number of flow instances should stay constant as long as Whiplash can quickly scan through the Whitelist. However, if there are excessive number of application patterns in the whitelist and flow instances are generated at an overwhelming rate, then Whiplash may not be able to find a match for a flow instance at all. For every flow instance in the PatternQueue, we can wait until the whitelist is scanned entirely for all other pending flow instances in the queue. However, this can further delay the pattern match procedure. Moreover, PatternQueue may crash with out-of-memory errors. Instead, we let Whiplash wait for maximum possible delay for a flow instance to find the fully matching subsequent flow instances. This however may yield many false negatives as the whitelist scanning may not complete before this wait time expires.

In case of TimedRETE it takes at most $\prod_{i=1}^{d}|C_{i}||WL_{C_{i}}|$ operations to check a full match for a flow instance, where |*C*_*i*_| is the number of child nodes at depth *i*, and $|WL_{C_{i}}|$ is the total number of entries in the whitelist of *C*_*i*_. It takes |*A*||*MS*| operations to retrieve the set of normal and abnormal time sequences, where |*A*| is the number of alpha nodes, and |*MS*| is the number of MatchStates entries in the alpha node. Similar to Whiplash, the number of flow instances waiting in the RETE network will be small as long as the WoT application behaves normally. However, TimedRETE is expected to perform better in case of excessive number of flow instances entering the system, as only the subset of the whitelist has to be checked through the RETE network.

## Evaluation

In this section we compare the performance of Whiplash and TimedRETE.

### Test environment

**Fig 16:**
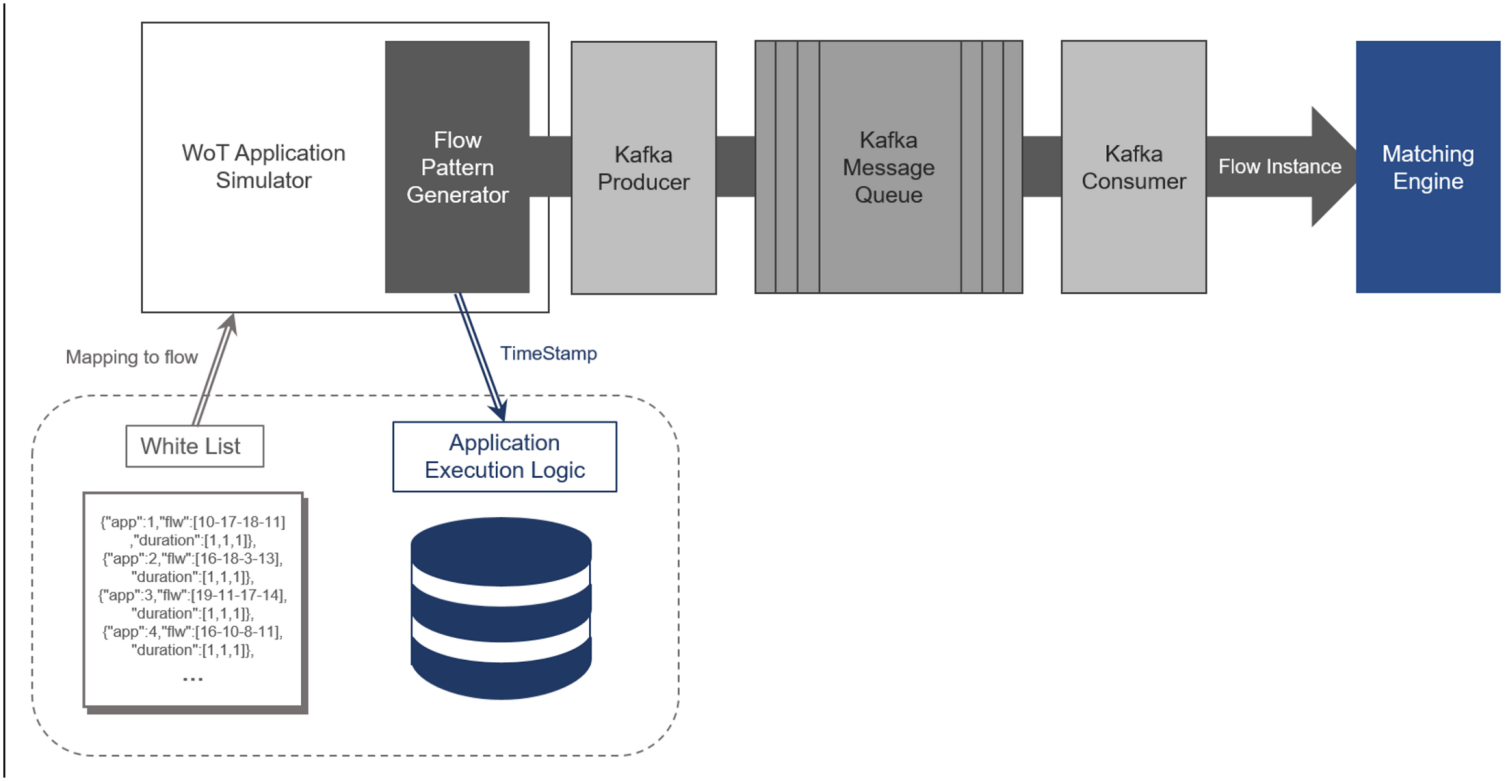
The testbed for performance measurement.

The test environment is set as shown in Fig 16.

The WoT Application Simulator (WAS) generates network flow instances. These flow instances are generated based on the randomly synthesized whitelist. WAS publishes the flow instances through Kafka which is a message queue that follows publish/subscribe communication paradigm [14]. The Matching Engine on the other end receives those flow instances through Kafka consumer. WAS logs the occurrence timestamp of every generated network flow instance into Application Execution Log. With this log, WAS can confirm the validity of a time sequence of flow instances that are identified by the Matching Engine.

Note that the executions of the randomly picked time sequence of network flows are interleaved, i.e., the flow instances can run concurrently. WAS randomly picks time delays between two flow instances from uniform distribution of time values ranging from 1 to 10 seconds. We also tried skewed distribution to time delays. However, the degree of skewness had no effect on the performance result.

Whiplash and TimedRETE running inside the Matching Engine are implemented in Java. We ran the test environment on a machine running Ubuntu 14.04 on Intel dual-core CPU @ 3.20 GHz with 4GB of RAM.

We measured the performance in terms of the memory usage and the point when an algorithm starts to see false negatives. False negatives are the cases when the Matching Engine falsely identifies a normal time sequence as an abnormal one. We also measured the average number of inquiries per flow instance that are issued to the WoT platform. This inquiry is to confirm whether a time sequence fully matching a whitelist entry is indeed valid according the Application Execution Log.

### Varying degree of flow overlap between applications

In this experiment, we vary the degree of network flow overlap among time sequences of network flows. We vary the degree by increasing the range of network flows to choose from 100 to 10,000, while the number of whitelist entries is fixed to 500. The inter-execution time between applications is fixed to 10 seconds. The inter-arrival time between abnormal flows is fixed to 1 second. The more network flows to choose from, the lower the degree of flow overlap gets. The less network flows to choose from, the higher the degree of flow overlap gets.

Table 1 shows the memory usage and the average number of inquiries (*I*) issued to the WoT platform whenever a fully-matching time sequence of flow instances is found by both algorithms under varying degree of flow overlap.

**Table 1:** The average number of inquiries to WoT platform and memory usage with varying range of network flows.

Algorithm		Whiplash		TimedRETE	
Parameter	Value	Memory Usage(MB)	*I*	Memory Usage(MB)	*I*
Range of network flows	100	32.51	0.2387	25.74	0.6543
	500	32.22	0.2389	28.74	0.3464
	1000	33.33	0.2391	29.78	0.3176
	5000	32.97	0.2392	30.63	0.3236
	10000	33.60	0.2389	31.24	0.3081

With a high degree of flow overlap (a low flow range), TimedRETE exhibits less usage of memory because many whitelist entries share the smaller set of aggregate nodes. With a low degree of flow overlap (a high flow range), TimedRETE exhibits higher usage of memory because it has to create more non-overlapping aggregate nodes. Whiplash consumes more memory than TimedRETE regardless of the varying degree value. Whiplash can consume up to 26% more memory with flow range of 100. This is because Whiplash has to unnecessarily populate a significant number of partial time sequences during the matching process.

In case of TimedRETE, *I* increases as the degree of overlap gets higher, i.e., flow range gets lower. With a smaller number of network flows to choose from, many whitelist entries overlap on a small number of aggregate nodes in the RETE network. In such a circumstance, TimedRETE issues more redundant inquiries to the WoT platform. In case of Whiplash, all of *I* are around 0.239. In Whiplash, *I* value does not change with the degree of flow overlap. This is because Whiplash does not store duplicate flows in a single node as in TimedRETE. Regardless of the redundancy, Whiplash always match a flow against the entire whitelist. The *I* value for Whiplash is relatively smaller at flow range of 100. This is because Whiplash asks the WoT platform once per whitelist entry when it finds the first matching full time sequence in PatternQueue.

### Varying size of whitelist

In this experiment, we vary the number of entries in the whitelist from 100 to 10,000. The range of network flows to choose from is fixed to 1,000. The inter-execution time between applications is fixed to 10 seconds and the inter-arrival time between abnormal flows is fixed to 1 second.

Table 2 shows the memory usage and the average number of inquiries (*I*) issued to WoT platform whenever a fully-matching time sequence of flow instances is found by both algorithms under varying size of whitelist.

**Table 2:** The average number of inquiries to WoT platform and memory usage with varying whitelist size.

Algorithm		Whiplash		TimedRETE	
Parameter	Value	Memory Usage(MB)	*I*	Memory Usage(MB)	*I*
Whitelist size	100	32.30	0.2435	27.17	1.6845
	500	33.33	0.2391	29.78	0.3176
	1000	35.32	0.2441	28.72	0.3315
	5000	Out of Memory	-	86.88	0.2781
	10000	Out of Memory	-	112.16	0.2545

TimedRETE tends to issue relatively more inquiries when the size of the whitelist is smaller. This is because there is a higher change of encountering a matched time sequence for most of the entries in the whitelist, as long as most application behave normally. Whiplash exhibits less number of inquires compared to TimedRETE, since many unrelated partial time sequences reside in PatternQueue. Also Whiplash is less scalable than TimedRETE as it fails to handle time sequence matching with the whitelist entries more than 1,000. With the same workload, TimedRETE can handles as many as 10,000 whitelist entries.

Whiplash starts to encounter excessive number of false negatives when the number of whitelist entries increases beyond 1,000. That is, Whiplash falsely identifies legitimate time sequences as abnormal patterns. TimedRETE on the other hand does not exhibit false negatives for the experiment with up to 10,000 whitelist entries. However, TimedRETE shows significant increase in memory when the number of whitelist entries is increased from 1,000 to 10,000. However, this result is a clear indication that TimedRETE is more scalable than Whiplash.

### Varying inter-execution time between applications

In this experiment, we vary the inter-execution time between applications from 1 second to 100 seconds. The range of flows to choose for composing an application is fixed to 1,000. The number of whitelist entries is fixed to 500. The inter-arrival time between abnormal flows is fixed to 1 second. As the interval of application execution increases, the number of generated flows decreases.

Table 3 shows the memory usage and the average number of inquiries (*I*) issued to WoT platform whenever a fully-matching time sequence of flow instances is found by both algorithms under varying inter-execution time between applications.

**Table 3:** The average number of inquiries to WoT platform and memory usage with varying inter-execution time between applications.

Algorithm		Whiplash		TimedRETE	
Parameter	Value	Memory Usage(MB)	*I*	Memory Usage(MB)	*I*
Inter-execution time between applications	1	Out of Memory	-	28.19	0.3213
	5	Out of Memory	-	29.98	0.3246
	10	33.33	0.2391	29.78	0.3176
	50	33.41	0.2393	28.83	0.3178
	100	30.22	0.1999	20.12	0.2668

In terms of the average inquiries (*I*) made to the WoT Platform per flow instance, TimedRETE issues more inquiries than Whiplash in all case similar to the previous experiments. The *I* value increases as the interval of application execution decreases. This is because the number of generated flows and matching throughput increase.

Beyond the inter-execution time of 5 seconds, Whiplash cannot handle any flow instances due to the excessive number of false negatives. TimedRETE uses less memory and can sustain up to the inter-execution time of 1 second i.e., around 75 incoming flow instances per minute. The stationary memory usage beyond the inter-execution time of 10 seconds is due to the fact that residence time of the flow instances in the RETE network is long. When more flow instances arrive at a faster rate (a low inter-execution time), they leave the RETE network quicker as the matching throughput increases as well. Our experiment shows that TimedRETE uses less than 30 MB of memory.

### Varying inter-arrival time between abnormal flows

In this experiment, we vary the proportion of abnormal patterns in the workload by changing the inter-arrival time between abnormal flows from 0.1 (0.38%) second to 10 seconds (27.6%). We also consider the case that abnormal flow instance is not generated at all (inter-arrival time of 0). The range of flows to choose for composing an application is fixed to 1,000, and the number of whitelist entries is fixed to 500. The inter-execution time between applications is fixed to 10 seconds. As the inter-arrival time between abnormal flows increases, the number of generated abnormal flows decreases.

Table 4 shows the memory usage and the average number of inquiries (*I*) issued to WoT platform whenever a fully-matching time sequence of flow instances is found by both algorithms under varying the inter-arrival time between abnormal flows.

**Table 4:** The average number of inquiries to WoT platform and memory usage with varying inter-arrival time between abnormal flows.

Algorithm		Whiplash		TimedRETE	
Parameter	Value	Memory Usage(MB)	*I*	Memory Usage(MB)	*I*
Inter-arrival time between abnormal flows	0	33.25	0.2483	29.61	0.3263
	10	32.62	0.2474	29.43	0.3275
	5	33.16	0.2464	29.12	0.3246
	1	33.33	0.2391	29.78	0.3176
	0.1	37.25	0.1796	29.26	0.2384

In terms of the average inquiries (*I*) made to the Platform per flow instance, TimedRETE issues more inquiries than Whiplash in all case similar to the previous experiments. The *I* value increase as the inter-arrival time between abnormal flows increases. This is because when the number of abnormal flows decreases, the proportion of normal flows increases, and the matching throughput increases as well.

TimedRETE uses less than 30 MB of memory regardless of the rate of abnormal flows. Whiplash periodically polls the WaitList of every alpha nodes to clear up flow instances whose validity is confirmed. Whiplash experiences increase in memory as the inter-arrival time decreases, i.e., the proportion of abnormal flow increases. In case of Whiplash, abnormal flow instances reside in the PatternQueue longer than the profiled delays of normal flow instances. In other words, Whiplash has to keep the abnormal flow instance in the PatternQueue until a full sequence of normal flows is detected. This leads to more memory usage by Whiplash than TimedRETE. In case of TimedRETE, every flow instance waiting in the WaitList is immediately removed whenever its *count* value decreases to 0. Therefore, TimedRETE does not wait until a full match is found.

## Related works

In this section, we put our work in the context of various related works.

Butun et al. presents the challenges and opportunities in anomaly detection for Cloud based IoT systems given the large number of heterogeneous connectivity and traffic patterns of IoT devices [15]. One of the challenges identified by this work is the identification of application-dependent behaviors in IoT data. In this paper, we point to the recent movement that several Web-based platforms such as IFTTT and Zapier provide means to mash up WoT (Web of Things) applications from a pool of heterogeneous Web services including sensors, actuators and data sources. Hence, we believe these WoT platforms are the source for gaining application awareness that can be utilized at the network monitor layer for detecting anomalies. Our cooperative framework between the WoT platforms and the network-layer anomaly detector can address the shortcomings of the existing works that do not leverage the application awareness as surveyed in [15, 16]. In [17], statistical wavelet analysis is conducted on Internet traffic data. Thottan and Ji focused more on analyzing IP network data [18]. More economical way of inferring sudden spikes in network traffic can be done with the summarized traffic data in Sketch [19]. Machine learning techniques can be also be used for detecting network intrusions [20, 21]. However, these statistical and AI-based approaches rely on the analysis based on the fragmented view of the network. None of these work attempted to take advantage of the mapping between between the network information and the application execution patterns. Therefore these works yield a significant number of false alarms in practice.

A few existing works use network flow information to detect intrusions [22–24]. Choi et al. devised a multi-pattern string matching on the packet payload to detect network intrusions [10]. These works only observe network-centric information. In contrast, we construct a whitelist of network flows out of the profiled behavior of WoT applications. Therefore, our work can monitor suspicious *application* activities at the network layer.

Kasinathan et al. studied intrusion detection techniques in the context of 6LoWPAN-based Internet of Things (IoT) [25]. This work focuses on the presentation of the reference architecture for detecting denial of service attacks on the IoT system. However, this work does not address the concern of faking an interaction between heterogeneous things on IoT/WoT application platforms. In this paper, we introduced a potential security breach by injecting false network flow instances to pretend that an application was executed as planned. Such security breach cannot be detected by either the application platform or the network monitoring agent independently. Our work presents a framework that facilitates the cooperation between both entities to detect such stealthy security threats by sharing detailed application execution patterns.

As we stated earlier in this paper, a study of the instrumentation of WoT applications is an orthogonal issue. However, we can consider employing profiling systems such as Magpie [26]. Magpie computes the performance of applications in terms of their usage of distributed computing resources at every stage in the application workflows. We can apply this technique to WoT platforms that manage applications composed of independently developed heterogeneous web services. We can focus more on adapting the system to generate network footprints (network flow instances), so that the footprints can be used as the whitelist for detecting anomalies at the network layer.

Lastly, there are a few off-the-shelve systems to process complex events based on RETE algorithms [11–13]. However, these systems are focused on matching events against a specific pattern. In our case, we have to retrieve all events that do not match a well-known event patterns for detecting anomalies. Doing so is more challenging especially when we have to consider the temporal information such as the known time delays between invocation of Web services in the WoT applications. In this paper, we implemented TimedRETE to tackle this issue.

## Conclusion

In this paper we presented a novel system that leverages the profiled application behavior from WoT platform in order to detect anomalies at the network layer. In the core of this framework lies an applied RETE-based matching engine that can detect abnormal network flow instances based on the application execution patterns made available by the WoT platforms. With this approach, administrators can interpret network flow information with regard to application logic. The administrators can use such contextual informations to detect and reason about abnormal behaviors more effectively. The experimental analyses show that our algorithm is tolerant to false detection and exhibits high scalability under reasonably configured application workloads. As a future work, we plan to study effective techniques for precisely profiling the behavior of the WoT platforms and deploy our network-layer anomaly detection system in the real-world setting.
